# Integrated RNA-seq and ATAC-seq analyses reveal muscle development differences between european meat pigeon and shiqi pigeon during embryonic to post-hatching stages

**DOI:** 10.3389/fcell.2026.1908892

**Published:** 2026-09-09

**Authors:** Yuanhao Han, Pufei Hong, Ziyang Liu, Tiande Chen, Kaiyue Chang, Weifeng Chen, Yirou Zhong, Ziyi Qiu, Zhihao Zheng, Xiujin Li, Zhongping Wu, Yayan Liang, Xumeng Zhang

**Affiliations:** 1 College of Animal Science & Technology, Zhongkai University of Agriculture and Engineering, Guangzhou, China; 2 Guangdong Engineering Technology Research Center of Biosafety and Intelligent Control for Aquatic Animals Diseases, Guangzhou, China

**Keywords:** ATAC-seq, embryonic pectoral muscle development, european meat pigeon, RNA-seq, shiqi pigeon

## Abstract

Poultry muscle growth and development critically depend on skeletal muscle formation during embryonic and hatching stages. However, early embryonic muscle development differences between pigeon breeds remain unclear. We used European meat pigeons and Shiqi pigeons, which differ in body size, as models. Pectoral muscles from both breeds were subjected to ATAC-seq and RNA-seq at embryonic day 14 (E14) and post-hatching day 1 (P1). At P1, Shiqi pigeons had a significantly greater proportion of myofibers with 1–50 μm diameters, higher myofiber density, and a predominant 50–100 μm distribution across developmental stages compared to European meat pigeons. Integrated ATAC-seq and RNA-seq analyses revealed developmental dynamics driven by muscle contraction, cardiac regulatory pathways, and PI3K-Akt/muscle remodeling signaling, as well as breed differences in chromosomal structure/cell cycle regulation, cancer-related pathways, metabolic homeostasis, and calcium/estrogen signaling. Furthermore, 62 chromatin accessibility-gene expression co-varying DEGs (59 upregulated) were enriched in insulin receptor binding and AMPK/cGMP-PKG pathways; key genes including *Sln, MYOM2, Trim63, Fabp3, ATP1A2, KLF15, IRS2*, and *RYR3* showed RT-qPCR expression highly consistent with RNA-seq (r ≥ 0.8181). This study found that the number of pectoral myofibers in Shiqi pigeons was significantly greater than in European meat pigeons at P1. Chromatin accessibility was higher near the transcription start site than in other regions. Integrating proximal, distal, and transcriptomic data, key candidate genes were screened and validated. Subsequently, genes differentially expressed between breeds were validated by tissue expression profiling, and *Sln* was selected for functional study. Cellular and quantitative experiments showed that *Sln* positively regulates pigeon embryonic myoblast proliferation and differentiation by upregulating *Myf5* during proliferation and *Myog* and *MyH1* during differentiation. Western blot confirmed higher MyoD and MyHC protein levels in *Sln*-overexpressing versus knockdown groups. This study revealed significant differences in pectoral myofiber diameter, embryonic chromatin structure, and gene expression between Shiqi and European meat pigeons, providing molecular insights into divergent muscle growth and development. The findings provide a theoretical basis for understanding growth performance differences among pigeon breeds and a foundation for molecular marker-assisted breeding.

## 1 Introduction

China’s pigeon farming industry has established the world’s largest production scale and is rapidly transitioning toward large-scale and automated operations (Jiang et al., 2019). Among the key local breeds driving this industry’s development, Shiqi pigeon stands out as a culinary specialty of Guangdong Province. Adult male Shiqi pigeons weigh 418–734 g, while females range 321–701 g. Squabs exhibit an average live weight of 585.61 g (Xu et al., 2024). The Mimas strain of European meat pigeon is a high-yielding white-feathered breed, with adult birds weighing 700–750 g. Squabs reach 610 g by 28 days of age. Its core competitive advantage lies in exceptional early-growth performance (Tang et al., 2018). Overall, Shiqi pigeon has lower body weight and slower growth rates than European meat pigeon. The specific regulatory pathways responsible for this disparity require further investigation.

Avian embryonic myogenesis constitutes a critical period for meat production potential, particularly as pectoral muscle development with the comprehensive formation of the skeletal muscle system. Generally speaking, animals with a higher number of myofibers exhibit a faster growth rate. In poultry, the count of myofibers, which is largely determined around the time of hatching, is closely correlated with muscle mass (Brooks, 2003; Brooks, 2003; Picard et al., 2010). Therefore, The development of skeletal muscle during embryogenesis up to hatching plays a crucial role in muscle growth. Muscle development originates from mesodermal somites, where myoblast differentiation is initially regulated by *Pax3/7* genes (Otto et al., 2006). Subsequently, the MRFs family genes, including *MyoD*, *Myf5*, *Myog*, and *MRF4*, initiate this process by activating the myogenic program and again play a crucial role in guiding the function of satellite cells for skeletal muscle regeneration. Within this established regulatory framework, core regulatory factors play a central role. These factors consist of two main groups: Pax3 and Pax7, and the myogenic regulatory factors (MRFs). The MRFs, including *Myf5, MyoD*, *myogenin*, and *MRF4*, are activated in a sequential manner to coordinate the determination, differentiation, and maturation of muscle cells (Geetha-Loganathan et al., 2005; Zammit, 2017). Multiple signaling pathways, such as Wnt, Shh, BMP, Notch, and FGF, interact to form a complex regulatory network during embryonic mesoderm formation, somitogenesis, and myogenic progenitor differentiation. The coordinated activity of these pathways contributes to modulating the ultimate count of myofibers (Dong et al., 2019; Geetha-Loganathan et al., 2005; Mao et al., 2023; Zammit, 2017).

In eukaryotic cells, DNA is compacted into chromatin through tight association with histones to form nucleosomes. The regulation of gene transcription is profoundly influenced by chromatin architecture, dynamic nucleosome positioning, and post-translational histone modifications. Crucially, chromatin accessibility governs the binding capacity of transcriptional regulators (e.g., transcription factors and enhancers), serving as a pivotal determinant for both gene activation and repression (Buenrostro et al., 2013). Recent advances in epigenomic profiling, particularly the high-resolution ATAC-seq technique, have revolutionized the study of chromatin accessibility landscapes. When integrated with RNA-seq data, ATAC-seq provides a powerful framework to dissect the molecular mechanisms underlying gene expression regulation (Buenrostro et al., 2015).

In a broader avian context, emerging research has begun to elucidate the epigenetic mechanisms governing muscle development. For instance, an integrated ATAC-seq and RNA-seq study in chickens characterized MYH1G-AS as a chromatin-associated lncRNA governing the development of skeletal muscle (Cai et al., 2024). Similarly, comparisons between fast- and slow-growing chicken breeds have uncovered genes critical to muscle-fat balance (Wang et al., 2024). Additionally, duck studies demonstrate temperature-dependent promoter methylation regulates myogenesis-associated gene expression (Wang et al., 2019), showing that epigenetic mechanisms are crucial for muscle growth in avian.

Our previous study investigating pectoral muscle development in European meat pigeon versus Shiqi pigeon during E14 and P1 stages revealed that Shiqi pigeon exhibited significantly higher myofiber density during early embryogenesis and a greater proportion of myofibers (1–50 μm diameter) at P1, with eight myogenesis-related differentially expressed genes identified (Li et al., 2023). While transcriptomic analyses in pigeons have delineated key regulators (e.g., MyoD) governing pectoral muscle development (Li et al., 2023; Mao et al., 2023; Wang et al., 2020). Current research on pectoral muscle development in pigeon embryos is limited, and the mechanisms involved remain incompletely elucidated.

This study systematically investigates the molecular regulatory mechanisms underlying pectoral muscle development from E14 to P1 using the European meat pigeon and Shiqi pigeon with significant differences in body size. Leveraging these results, this study seeks to explore the mechanisms underlying the divergent developmental patterns between Shiqi and European meat pigeons during embryonic and postnatal stages. To achieve this, we first performed H&E staining to observe morphological characteristics. Integrated multi-omics analysis (RNA-seq and ATAC-seq) was then conducted on pectoral muscle tissues from both pigeon breeds to uncover the dynamic interplay between chromatin accessibility and transcriptional regulation, and to identify key epigenetic and transcriptional networks driving muscle development. Subsequently, we examined the tissue expression profiles of these key genes across various pigeon tissues, validated protein expression by Western blot, and functionally confirmed the findings in pigeon embryonic myoblasts. Therefore, this research will provide a theoretical foundation for systematically insight into the molecular underpinnings of skeletal muscle developmental differences in pigeon breeds of varying body sizes and support the future development of molecular marker-assisted breeding strategies.

## 2 Article types

This manuscript is submitted as an Original Research article. It presents novel, hypothesis-driven investigations into breed-specific differences in pigeon pectoral muscle development, integrating ATAC-seq and RNA-seq at two developmental stages (embryonic day 14 and post-hatching day 1). The study includes rigorous experimental validation (RT-qPCR, myoblast proliferation/differentiation assays, Western blot) and functional characterization of the candidate gene *Sln*. The work provides comprehensive molecular insights into chromatin accessibility, gene expression, and muscle growth regulation, fitting the scope of Frontiers in Cell and Developmental Biology–Chromosome and Chromatin Biology. The manuscript complies with the journal’s requirements for Original Research.

## 3 Materials and methods

### 3.1 Animal ethics

The animal experiments complied with the China Council on Animal Care guidelines and obtained approval from the Animal Experiment Committee of Zhongkai University of Agriculture and Engineering (Approval No. 2021112709).

### 3.2 Animals and tissue sampling

To analyze pectoral muscle development, samples were obtained from European meat and Shiqi pigeons at the embryonic (E14) and early post-hatching (P1) stages. For each breed and stage, three biological replicates (n = 3) were collected, totaling 12 pigeons. The carcass performance traits of the two breeds are provided in Supplementary Table S1. The left pectoral muscles were fixed in paraformaldehyde for histological examination. Samples taken from the right side were promptly flash-frozen in liquid nitrogen, then moved to a −80 °C freezer for keeping. These cryopreserved samples were then designated for multi-omics analysis, encompassing RNA-seq, ATAC-seq, and targeted gene expression profiling.

### 3.3 Hematoxylin–Eosin (H&E) staining of pectoral muscle

The histological characteristics of pectoral muscle fibers in Shiqi and European meat pigeons were obtained from our previously published study (Li et al., 2023). Briefly, the previously reported H&E staining images and morphometric analyses, including myofiber diameter distribution, myofiber density, and estimated total myofiber number, are presented in Supplementary Figure S1 as background information to illustrate breed-specific differences in pectoral muscle development. Detailed procedures for tissue preparation, histological staining, image acquisition, and morphometric measurements have been described previously.

### 3.4 RNA-seq

Pectoral muscle samples were first harvested, with three replicates collected per breed at each developmental stage. Total RNA extraction followed, using TRIzol reagent (Invitrogen, USA) per the manufacturer’s protocol. The isolated sample then underwent quality assessment. An assessment of RNA integrity was performed. The first step involved determining the RNA Integrity Number (RIN). This measurement used an Agilent 2100 Bioanalyzer system (Agilent Technologies, United States). The second step confirmed overall integrity via agarose gel electrophoresis. All electrophoresis steps were carried out under RNase-free conditions. For library construction, mRNA was enriched via Oligo (dT) beads and subjected to fragmentation into pieces of 200–300 nucleotides. In line with the manufacturer’s guidelines for utilizing the NEBNext Ultra RNA Library Prep Kit (NEB #7530, United States), the fragmented mRNA was first reverse-transcribed to create double-stranded cDNA. Following this, the cDNA underwent a standard workflow including end repair, adenylation, and finally, adapter ligation for Illumina sequencing. Hieff NGS® DNA Selection Beads were utilized for library processing. With these beads, purification and size selection (250–350 bp) ensured fragment integrity. PCR amplification came next, enriching the libraries for downstream use. An Illumina NovaSeq X Plus platform (supplied by Gene *Denovo* Biotechnology Co., China) then performed sequencing. This high-throughput analysis generated 150 bp paired-end reads from the resulting libraries.

Fastp v0.18.0 was employed for raw sequencing data, a step targeting the removal of adapter-containing reads as part of quality control, reads containing >10% ambiguous nucleotides (N) or exhibiting poor quality (>50% bases with Q-score ≤20) (Chen et al., 2018). Quality-passed reads were then aligned to an rRNA (ribosomal RNA) database using Bowtie2 v2.2.8 (Yu et al., 2015), and rRNA-aligned sequences were excluded. Remaining reads were subsequently mapped to the *Columba livia* reference genome assembly GCA_000337935.2 (NCBI accession GCA_000337935.2; accessed 13 December 2017) via HISAT2 v2.1.0 (Kim et al., 2015), followed by transcript reconstruction with StringTie v1.3.1 (Pertea et al., 2015). Gene expression was quantified as FPKM.

Sample relationships were evaluated via correlation analysis and PCA using R packages. Genes differentially expressed (DEGs) were detected using DESeq2 (Love et al., 2014) and edgeR (Robinson et al., 2009) (FDR <0.05, |fold change| ≥ 2). Functional enrichment analyses (GO, KEGG, Disease Ontology, Reactome) were performed using hypergeometric tests with FDR correction (Shannon et al., 2003).

### 3.5 ATAC-seq

Three biological replicates were processed by Guangzhou GeneDenovo Biotechnology Co., Ltd. following a standardized protocol. Nuclei were isolated using hypotonic lysis buffer, and chromatin fragmentation was performed via Tn5 transposase (37 °C, 30 min) to simultaneously cleave open chromatin regions and ligate sequencing adapters. Transposed DNA required purification before downstream use. A QIAGEN MinElute Kit was employed for this step. Next, the purified DNA was amplified via 12-cycle PCR. The final output of this amplification was libraries (Buenrostro et al., 2015). Size selection (150–800 bp fragments) was achieved via magnetic bead purification, The Illumina NovaSeq X Plus platform, a commercially available system, was applied to perform paired-end sequencing (PE150) under standard protocol.

Data processing began with quality control using FastQC (v0.11.9) and the removal of reads which were adapter-contaminated, of low quality (>50% bases with Q-score≤20), or contained >10% undefined nucleotides. The clean reads were then aligned against the rock pigeon reference genome (GCA_000337935.2) with Bowtie2 (v2.2.8), employing the sensitive-local parameters for alignment (Kim et al., 2015; Langmead and Salzberg, 2012), with mitochondrial/chloroplast sequences excluded and duplicates removed via SAMtools (v1.12).

Open chromatin peaks were identified using MACS2 (v2.1.2),retaining regions with Q-value <0.05. Peaks were annotated to regulatory elements (promoters, enhancers, *etc.*) via ChIPseeker (v1.30.3), and differential accessibility between groups was analyzed using DiffBind (FDR <0.05). Enrichment of peak-linked genes in biological pathways was statistically validated via concurrent GO term and KEGG pathway mapping (FDR ≤0.05) using hypergeometric testing (Yu et al., 2015; Zhang et al., 2008).

### 3.6 Integrated analysis of ATAC-seq and RNA-seq

To pinpoint critical genes potentially under chromatin-mediated regulation, the overlap between RNA-seq DEGs and ATAC-seq DAR-associated genes was determined in R (v4.3.1) using the VennDiagram package (v.1.7.3). To functionally characterize these overlapping genes, we performed GO and KEGG enrichment analysis using the OmicShare online tool (https://www.omicshare.com/tools/home/soft/getsoft.html). Furthermore, Spearman’s correlation indices were calculated using OmicShare tools to assess additional relationships (http://www.omicshare.com/tools).

### 3.7 Gene functional annotation

Gene Ontology (GO) enrichment analysis for DEGs was conducted in R (v4.3.1) utilizing the Bioconductor package (Huber et al., 2015). The analysis employed a hypergeometric test, as detailed in Sections 2.5–2.6. GO terms for the reference genome were retrieved from the corresponding annotation file. Significantly enriched terms (FDR ≤0.05 using the Benjamini–Hochberg correction) were identified, and semantic redundancy was subsequently reduced by pruning based on hierarchical ontology relationships.

To further investigate the functional interactions, PPI (protein-protein interaction) network was constructed via the STRING database and displayed in Cytoscape version 3.7.1. This analysis was complemented by a pathway enrichment analysis employing KEGG (the Kyoto Encyclopedia of Genes and Genomes), which was conducted using the hypergeometric test with a significance cutoff of FDR ≤0.05 and a minimum requirement of five genes per pathway. Both analyses consistently applied the statistical thresholds (|fold change| ≥ 2 and FDR correction) specified in the original workflow (Shannon et al., 2003).

### 3.8 Gene expression analysis

Using TRIzol reagent (OMEGA, United States; Genstar, China), total RNA was purified from tissue samples for each breed in three biological replicates, and cDNA was synthesized with a commercial first-strand kit (Takara, China). adhering to the respective manufacturers’ protocols. Quantitative real-time PCR (qPCR) assays were conducted in technical triplicates on a LightCycler 480 II system (Roche, Switzerland) using a SYBR Green kit (GenStar, China). Gene expression quantification was carried out using qPCR employing gene-specific primers (Supplementary Table S4, designed with Primer 5.0) and Ct values were first calibrated to GAPDH, a housekeeping gene serving as the internal control. For fold change analysis, the 2^(-ΔΔCT)^ approach was then utilized. This entire workflow followed a protocol: 95 °C for 1 min (initial denaturation), succeeded by 40 cycles (95 °C 10 s, 60 °C 34 s) to drive amplification. GraphPad Prism 8.0 was the selected software for statistical work. It supported all analytical tasks in this study. Two-way ANOVA, paired with Tukey’s test for *post hoc* comparisons, was one method. Additionally, correlation coefficients (r) were computed to quantify the relationship between target gene expression levels (quantified via 2^(-ΔΔCT)) and breed classifications.

### 3.9 Pigeon embryonic myoblast isolation

Primary myoblasts were isolated from European meat pigeon embryos (E14) provided by Meizhou Jinlv Modern Agricultural Development Co., Ltd. Briefly, embryos underwent surface disinfection using 75% ethanol followed by UV irradiation for 15 min. Under aseptic conditions, the pectoral muscle tissues underwent dissection and washed with PBS, and minced. The tissue fragments were homogenized and digested with 0.25% trypsin (GIBCO) at 37 °C for 5–10 min. The digestion was stopped by introducing the same volume of DMEM/F12 medium supplemented with 20% FBS (GIBCO). The homogenate was passed through a 70 μm cell strainer and then spun at 1000 × g for 5 min. Complete medium, consisting of DMEM/F12, 20% FBS, and 1% antibiotics, was used to resuspend the cell pelle, adjusted to a density of 5 × 10^5^ cells/mL, and seeded. After a 1.5–2 h pre-plating step at 37 °C under 5% CO_2_, the supernatant containing predominantly myoblasts was collected and transferred for further expansion.

### 3.10 EdU

Cells were pulse-labeled with 50 μM EdU (prepared by diluting reagent A 1:1000 in complete medium) for 2 h. Following incubation, EdU-containing medium was discarded, followed by a single PBS wash prior to fixing with 4% paraformaldehyde dissolved in PBS at room temperature for 30 min. Post-fixation, cells were treated with a 2 mg/mL glycine solution (50 μL) for 5 min on a shaker to quench residual fixative. The cells were then rinsed twice washed with PBS (5 min per wash) and then placed into 0.5% Triton X-100 in PBS on a shaker for 10 min to achieve permeabilization, before being washed once more with PBS.

Following the EdU labeling and fixation steps, the Apollo staining reaction was performed. Briefly, cells were exposed to 100 μL of 1× Apollo staining reaction solution (for a 1 mL system: 938 μL deionized H_2_O, 50 μL reagent B, 10 μL reagent C, 3 μL reagent D, and 9 mg reagent E) for 30 min on a shaker at room temperature, protected from light. Staining solution was subsequently withdrawn, followed by washing the cells two to three times for 10 min each with permeabilization buffer, and then a 5-min wash with PBS. For nuclear counterstaining, nuclear staining was performed by incubating the cells with 100 μL of 1× Hoechst 33,342 solution. (prepared by a 100:1 dilution of reagent F in deionized H_2_O) for 30 min under light-protected conditions. After 1–3 subsequent washes with PBS, the samples were preserved under 100 μL of PBS. The quantification of EdU-positive cells was performed on images acquired with an inverted fluorescence microscope, using ImageJ software (v6.0).

### 3.11 Immunofluorescence

Culture medium was suctioned away, and the cells were subjected to 2–3 PBS washes. A 10-min fixation step at room temperature with 4% paraformaldehyde under gentle shaking was then performed. This was followed by three 5-min PBS washes. Permeabilization was achieved using a 10-min treatment using 0.5% Triton X-100 at ambient temperature, preceding another three PBS washes. Nonspecific binding was prevented by a 1 h room temperature blockage with 5% BSA.

Cells were incubated overnight at 4 °C with a primary antibody against myosin heavy chain (MyHC; M4276, Shanghai Abways Biotechnology Co., Ltd., Shanghai, China; dilution 1:200). The cells were then incubated for 1 h at room temperature in the dark with a fluorescently labeled goat anti-rabbit IgG secondary antibody (Wanlei Biotechnology, Shenyang, China; catalog number: WLA023a; dilution 1:500). Both antibody incubation steps were followed by three PBS washes. Both antibody steps were performed in the dark, with three PBS washes conducted after each incubation. Following a final series of three PBS washes, DAPI was used for nuclear counterstaining at room temperature for 5 min in light-protected settings. Subsequently, Images were obtained, this was achieved using an inverted fluorescence microscope (Olympus, Tokyo, Japan, IX73P1F). The MyHC-positive rate and myotube diameter were quantified using ImageJ software (v6.0). To ensure reproducibility, a consistent reagent volume of 100 μL per well and precise timing were maintained throughout the procedure.

### 3.12 Over-expression and the RNA interference assays

This study comprised four experimental groups: an over-expression group, its corresponding empty vector control, an RNAi group (siRNA-*Sln*), and its negative control (siRNA-NC). The over-expression plasmids (pcDNA3.1-*Sln*) and the empty vector control (pcDNA3.1-NC) were commercially sourced from Qingdao Biotechnology Co., Ltd (see Supplementary Table S16 for vector sequence). For transfection, pigeon embryonic myoblasts were plated into 24-well plates and grown until they reached 70%–80% confluency. Following the protocol for Lipofectamine 3000 (Thermo Fisher Scientific), in Opti-MEM medium, complex formation was achieved by incubating a mixture of plasmid DNA and reagent (2 µL per µg DNA) for 15 min at ambient temperature. The complexes were subsequently introduced dropwise to the cells. Following a 6 h interval, the transfection medium was swapped with fresh proliferation medium to enable subsequent analysis.

Three pairs of siRNA-*Sln* and a non-targeting control siRNA (siRNA-NC), both sourced from Qingdao Biotechnology Co., Ltd., were delivered into pigeon embryonic myoblasts. The primer information for the three pairs of siRNA-*Sln* is provided in Supplementary Table S16. This transfection employed Lipofectamine 3000 (Invitrogen) per the manufacturer’s guidelines. Cells were seeded into 24-well plates for the procedure. The transfection used a complex prepared by mixing 30 μmol of siRNA with 10 µL of Lipofectamine 3000 in Opti-MEM medium (Gibco). The medium was refreshed with complete medium at the 6 h time point. Cells were then processed for subsequent mRNA or protein analysis 48 h post-transfection. Each transfection experiment included three independent biological replicates.

### 3.13 Western blotting

Total proteins were separated using SDS-PAGE and then moved to PVDF membranes. Subsequently, the membranes were treated with 5% skim milk dissolved in PBST at room temperature for 1 h to block non-specific binding. Following this, the membranes were exposed overnight at 4 °C to primary antibodies listed below; anti-SLN (1:1000, Proteintech, 18395-1-AP), anti-MyoD diluted at 1:1000 (Abcam, ab16148), anti-MyHC at 1:10,000 dilution (Sigma, M4276), and anti-GAPDH diluted at 1:10,000 (Abcam, ab181602). Subsequently, An incubation step was performed by subjecting the membranes to Suitable HRP-linked secondary antibodies were applied for 1 h at ambient temperature (mouse-specific, a 1:10,000 dilution, Abcam, ab97023; alternatively rabbit-specific, a 1:10,000 dilution, Wanlei, WLA32023a). For visualization, protein bands were visualized using the chemiluminescence (ECL) kit (Beyotime) and documented on a Tanon-200 multi imaging system. The relative intensity of the bands was then quantified through densitometry with ImageJ software (Version 1.8.0). Three separate replicate experiments were performed.

### 3.14 Statistical analysis

All data are presented as the mean ± SEM from at least three independent biological replicates. Statistical analyses were performed using GraphPad Prism 8.0 (GraphPad Software, San Diego, CA, USA). Comparisons between two groups were performed using an unpaired two-tailed Student’s t-test, whereas comparisons among multiple groups were analyzed using one-way analysis of variance (ANOVA) followed by Tukey’s multiple comparison test. Differences were considered statistically significant at *P* < 0.05.

## 4 Results

### 4.1 Analysis of pectoral muscle H&E staining results of two pigeon breeds

Previous studies have demonstrated that Shiqi pigeons and European meat pigeons exhibit distinct pectoral myofiber characteristics during early development. Specifically, Shiqi pigeons showed higher myofiber density and a greater proportion of smaller-diameter fibers at hatching compared with European meat pigeons ((Li et al., 2023); Supplementary Figure S1,Supplementary Tables S1-S3). However, the molecular mechanisms underlying these breed-specific differences in muscle development remain unclear. Therefore, we performed integrated transcriptomic and epigenomic analyses to identify candidate genes and regulatory pathways associated with pigeon pectoral muscle development.

### 4.2 Transcriptomic profiling identifies key genes and pathways associated with pigeon pectoral muscle development

Following the QC of sequencing data obtained from 12 samples, all samples exhibited Q20 scores exceeding 97.28% and Q30 scores surpassing 92.67%. Alignment analysis against the reference genome revealed that 75.79%–81.49% of clean reads were successfully mapped (Supplementary Tables S5-S7; Supplementary Figure S2).

**FIGURE 1:**
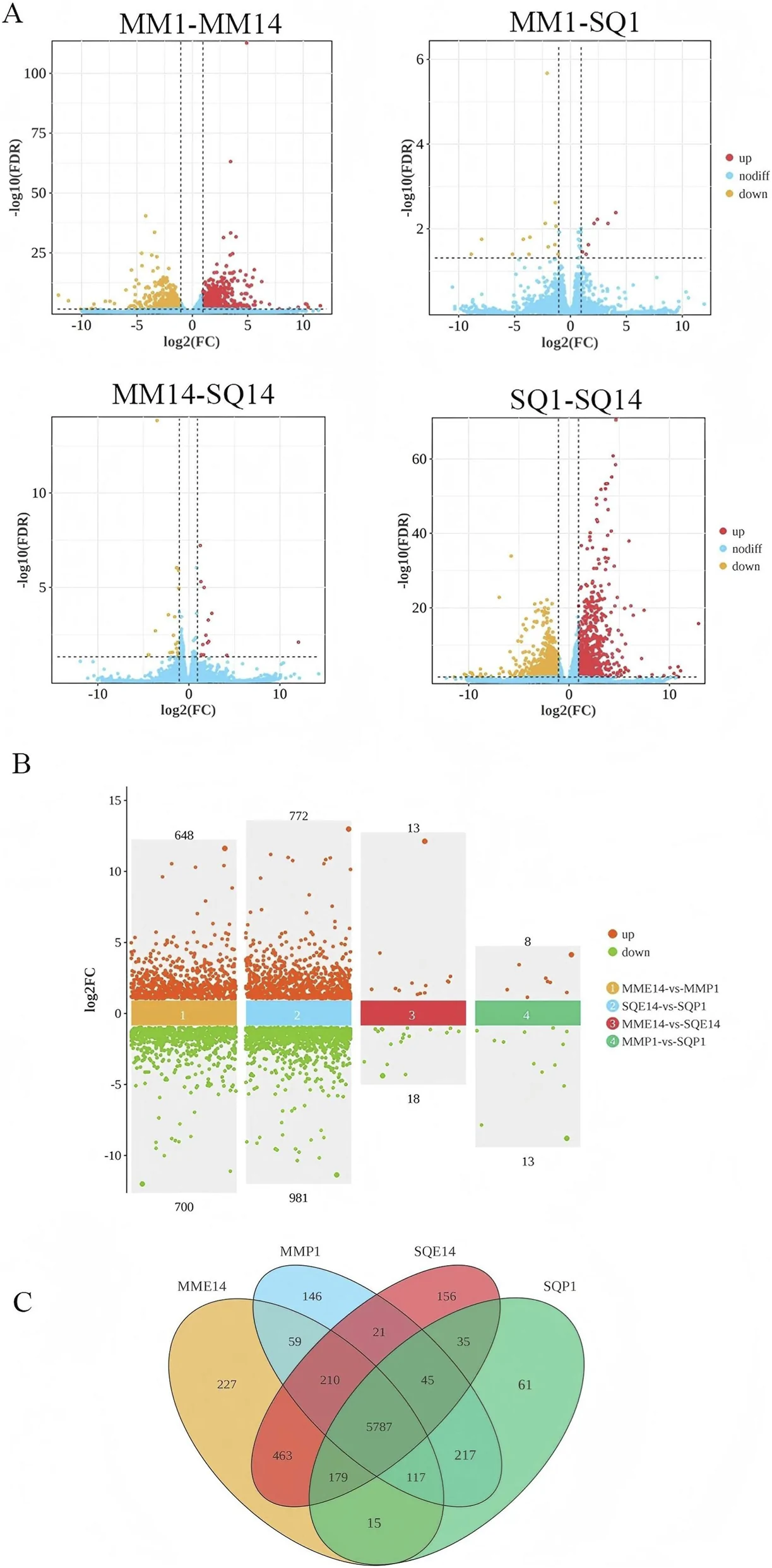
Transcriptomic profiling and functional convergence of differential gene expression patterns. **(A)** Volcano plot of DEGs; **(B)** Numbers of up- and downregulated DEGs identified in each of the four pairwise comparisons (MME14 vs. SQE14, SQE14 vs. SQP1, MME14 vs. MMP1, and MMP1 vs. SQP1). DEGs were identified separately for each pairwise comparison rather than across all four groups simultaneously; **(C)** Breed- and stage-specific DEGs overlap between European meat and Shiqi pigeons.

Differential expression analysis was conducted across the four groups, which were structured by the two factors of breed and age, to identify DEGs. The resulting DEGs were preliminarily visualized in a volcano plot (Figure 1A). Differential expression analysis was performed through four pairwise comparisons (MME14 vs. SQE14, SQE14 vs. SQP1, MME14 vs. MMP1, and MMP1 vs. SQP1). Integrative analysis of the breed comparisons identified 755 breed-associated DEGs, whereas integration of the developmental-stage comparisons identified 1,118 stage-associated DEGs (Figure 1B), while overlapping DEGs were further characterized (Figure 1C; Supplementary Table S8).

**FIGURE 2:**
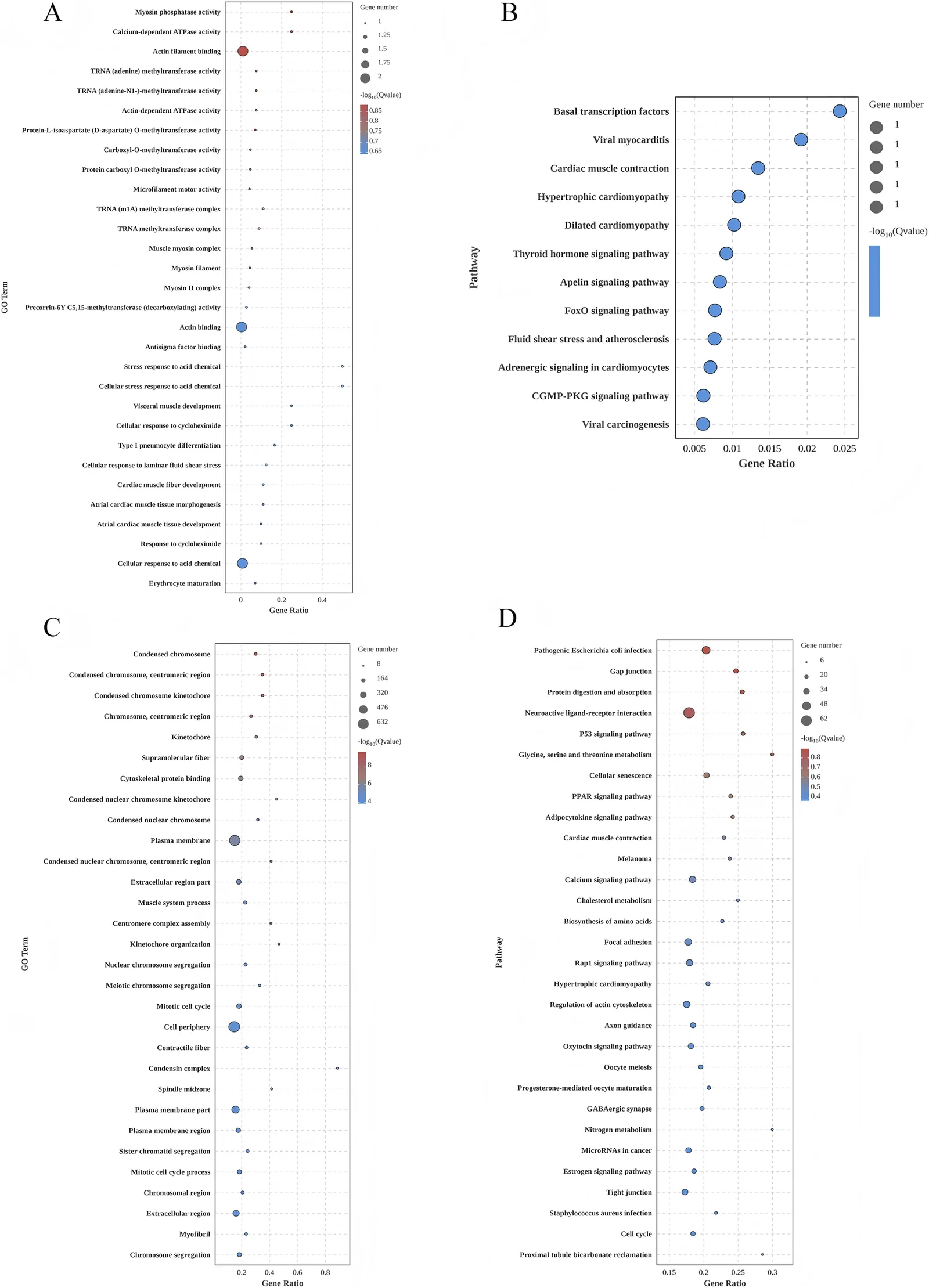
Functional enrichment analysis associated with muscle development dynamics and breed divergence. **(A)** GO Enrichment Analysis (Developmental stage comparison: E14 vs. P1); **(B)** KEGG Pathway Enrichment Analysis (Developmental stage comparison: E14 vs. P1); **(C)** GO Enrichment Analysis (Breed comparison: MM vs. SQ); **(D)** KEGG Pathway Enrichment Analysis (Breed comparison: MM vs. SQ).

Across developmental stages and between breeds, GO and KEGG enrichment analyses revealed distinct functional profiles. Developmental analysis highlighted significant enrichment for molecular functions involved in muscle contraction (e.g., myosin phosphatase activity, calcium-dependent ATPase activity, actin binding, myosin complex organization) (Figure 2A) and pathways related to cardiac muscle function/regulation (e.g., hypertrophic/dilated cardiomyopathy, cardiac muscle contraction, thyroid hormone signaling, adrenergic signaling) (Figure 2B), suggesting DEGs may primarily impact muscle cell contraction and energy metabolism. Conversely, at the inter-breed level, GO analysis implicated genes in chromosomal structure/cell cycle regulation (e.g., condensed chromosome, mitotic cell cycle; Figure 2C), while KEGG pathways pointed to roles in cancer (e.g., melanoma, p53 signaling), metabolism (e.g., cholesterol/amino acid), and cellular signaling (e.g., calcium, estrogen signaling; Figure 2D; Supplementary Tables S8-S9), suggesting breed differences involve coordinated genomic integrity, proliferation, and metabolic homeostasis. RNA-seq revealed developmental-stage DEGs enriched in muscle contraction functions and cardiac pathways, whereas breed-biased DEGs were enriched in chromosome/cell cycle regulation, cancer pathways, and metabolic homeostasis signaling.

### 4.3 ATAC-seq profiling identifies dynamic chromatin accessibility changes during pectoral muscle development

**FIGURE 3:**
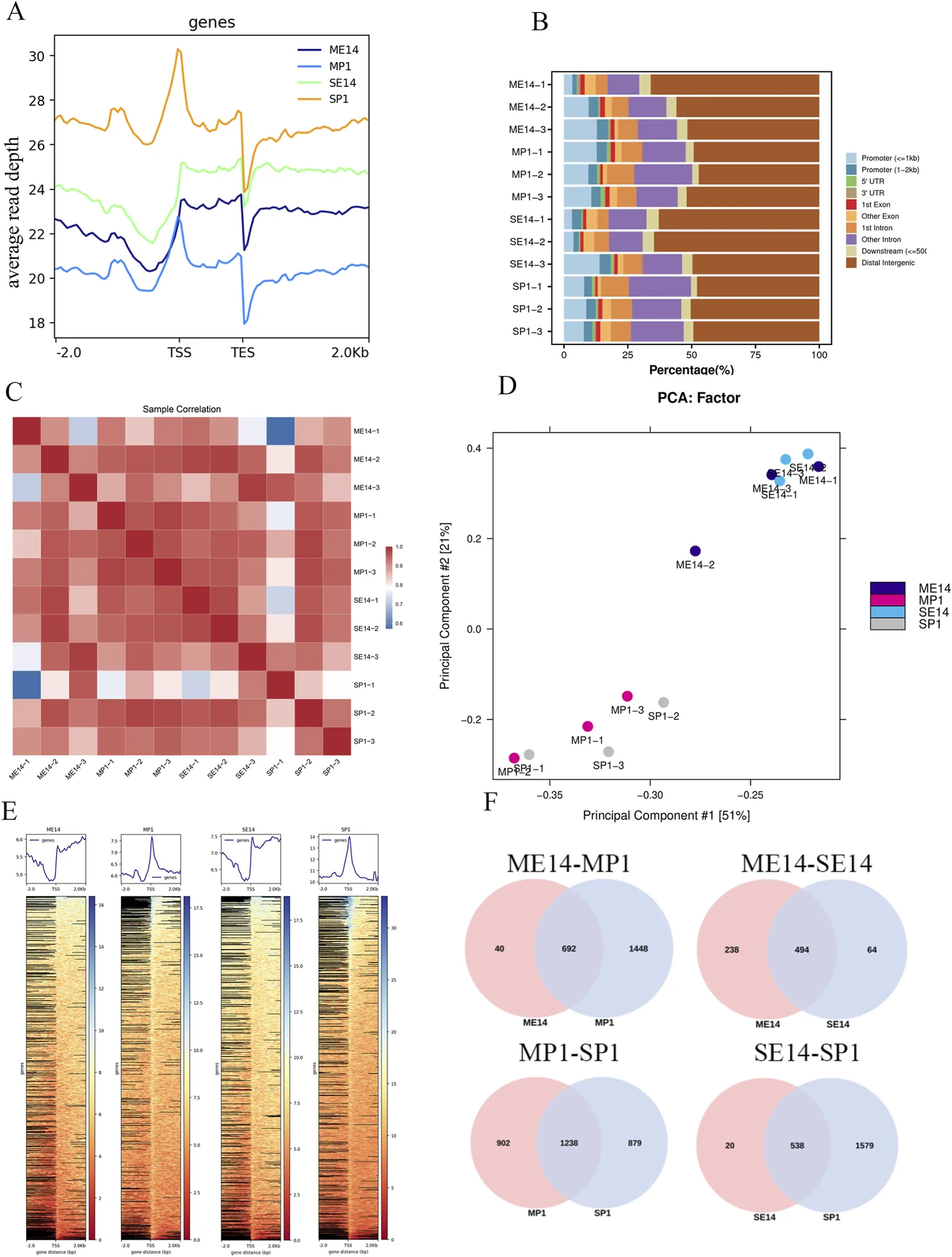
Quality control metrics for ATAC-seq data integrity and reproducibility. **(A)** Enrichment of ATAC-seq signals around TSS regions; **(B)** Distribution of functional genomic regions; **(C)** Correlation heatmap between samples; **(D)** Principal Component Analysis (PCA) plot; **(E)** ATAC-seq signal enrichment plot (±2 kb around peak centers); **(F)** Differential peak analysis.

To reveal the mechanistic basis of pectoral myofiber differences between European meat pigeons and Shiqi pigeons, ATAC-seq analysis was conducted to characterize genome-wide chromatin accessibility patterns. A total of 1,056, 758, 776 raw reads were obtained, and the same number of clean reads were retained after quality filtering (Supplementary Tables S10–S11). The distribution analysis of accessible chromatin regions showed that the majority of ATAC-seq peaks were located around transcription start sites (TSSs), particularly within 2 kb upstream and downstream of TSSs (Figures 3A,B), indicating that these accessible regions may be involved in transcriptional regulation. The enrichment pattern of accessible regions around TSSs and transcription termination sites (TESs) further demonstrated similar genomic distribution characteristics among biological replicates (Figure 3E). Pairwise Pearson correlation analysis based on ATAC-seq peak signals showed high correlation among biological replicates, confirming the reliability and reproducibility of the sequencing data (Figure 3C; Supplementary Tables S12–S14). Principal component analysis (PCA) further revealed clear separation between European meat pigeons and Shiqi pigeons, while biological replicates within each group showed relatively consistent clustering (Figure 3D). In addition, comparative analysis of accessible regions between groups identified both shared and breed-specific chromatin accessible regions, highlighting differences in genome-wide chromatin landscapes between European meat pigeons and Shiqi pigeons (Figure 3F). These results collectively demonstrate the high quality of ATAC-seq data and provide a reliable basis for subsequent analysis of breed-specific regulatory mechanisms.

**FIGURE 4:**
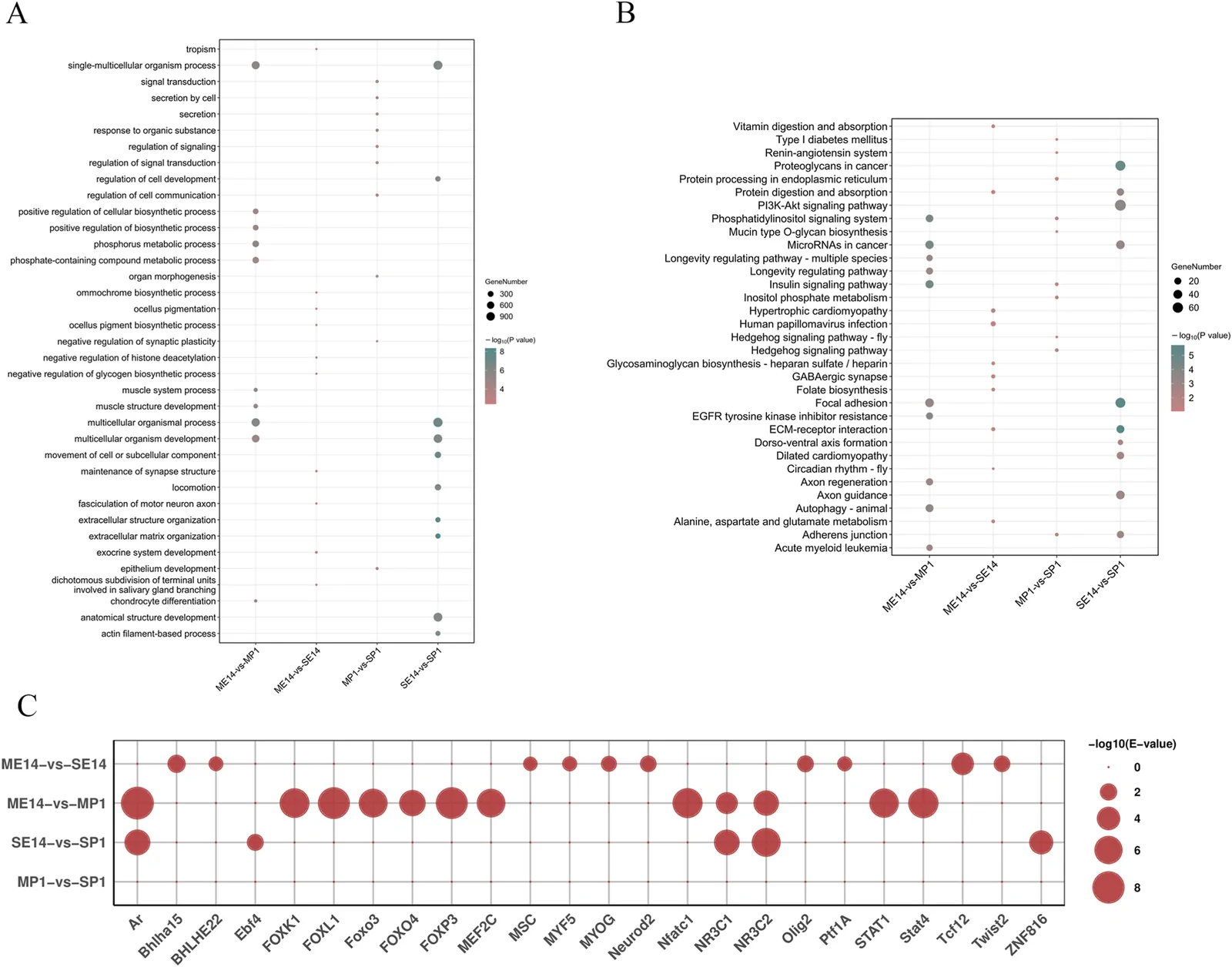
Functional annotation and chromatin accessibility associations. **(A)** GO enrichment analysis of top 20 significant biological processes; **(B)** KEGG pathway analysis of top 15 enriched pathways; **(C)** Enrichment analysis of genes associated with differentially accessible chromatin regions.

Combined GO and KEGG enrichment analyses across developmental stages and between breeds revealed distinct functional characteristics associated with chromatin accessibility changes. During developmental transitions, GO enrichment analysis identified biological processes related to developmental regulation, muscle system processes, extracellular matrix organization, and metabolic adaptation (Figure 4A; Supplementary Table S15). KEGG enrichment analysis further revealed that development-associated accessible regions were mainly involved in pathways related to muscle function and remodeling, including hypertrophic cardiomyopathy, dilated cardiomyopathy, cardiac muscle contraction, PI3K-Akt signaling, renin-angiotensin signaling, and insulin signaling pathways (Figure 4B; Supplementary Table S16). These results suggest that developmental changes in pectoral muscle are accompanied by dynamic regulation of chromatin accessibility associated with muscle function, tissue remodeling, and metabolic regulation.

In contrast, comparative analysis between European meat pigeons and Shiqi pigeons revealed breed-specific enrichment patterns. GO analysis showed that differential accessible regions were associated with chromosome organization, cell cycle regulation, and cellular proliferation-related processes (Figure 4C). KEGG analysis further indicated enrichment in pathways related to genomic stability, cancer-associated signaling, metabolic regulation, and cellular signaling pathways, suggesting that breed-specific differences in pectoral muscle development may involve coordinated regulation of chromatin structure, proliferative capacity, and metabolic adaptation.

### 4.4 Integrated ATAC-seq and RNA-seq analysis uncovers candidate regulatory genes driving muscle development

**FIGURE 5:**
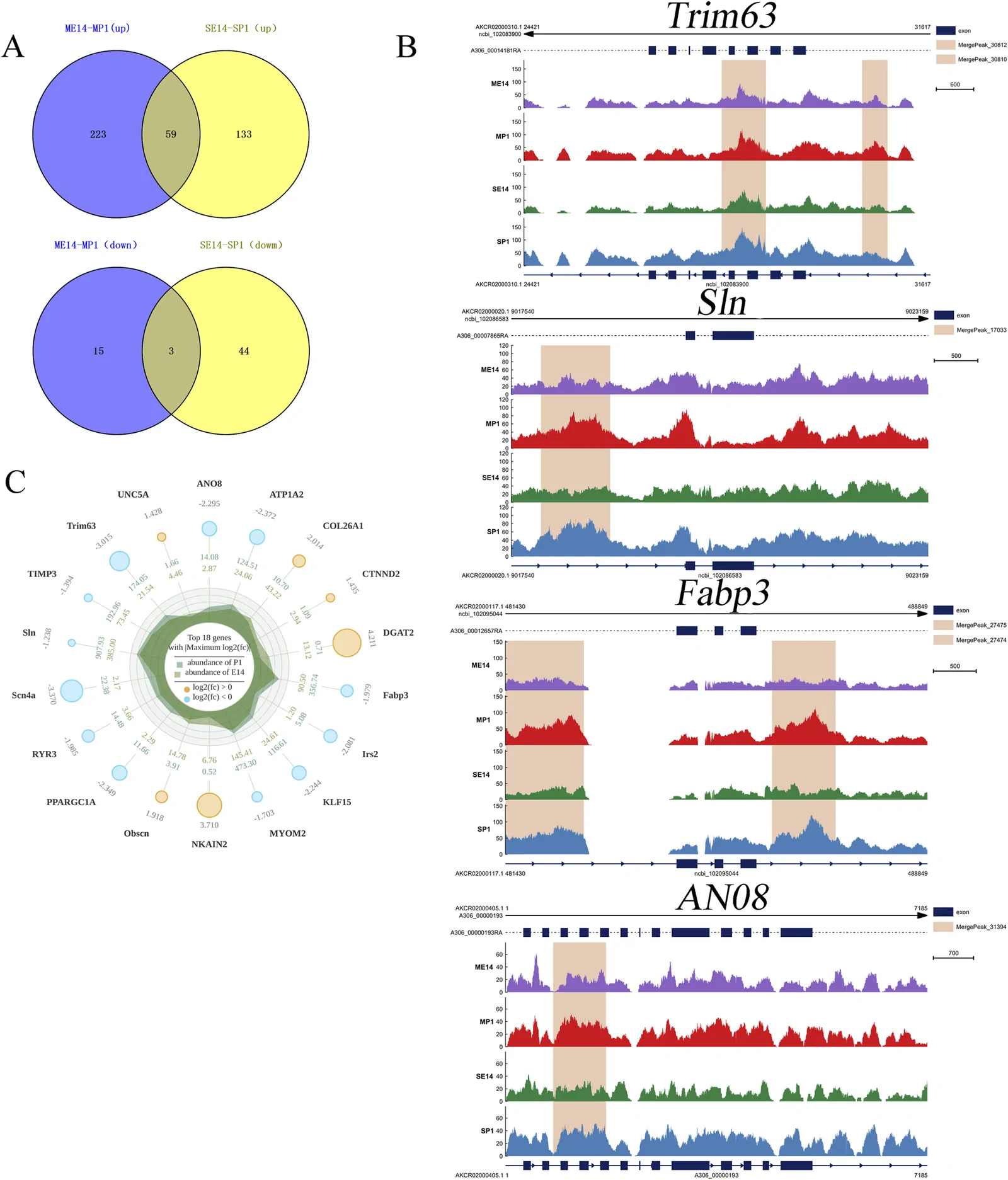
Integrated analysis of ATAC-seq and RNA-seq data. **(A)** Comparative visualization of RNA-seq (left) and ATAC-seq (right) results; **(B)** Genomic peak diagram illustrating chromatin accessibility and gene expression relationships (Exon regions represent transcriptome coding sequences); **(C)** Radar map of the top 18 candidate genes.

**FIGURE 6:**
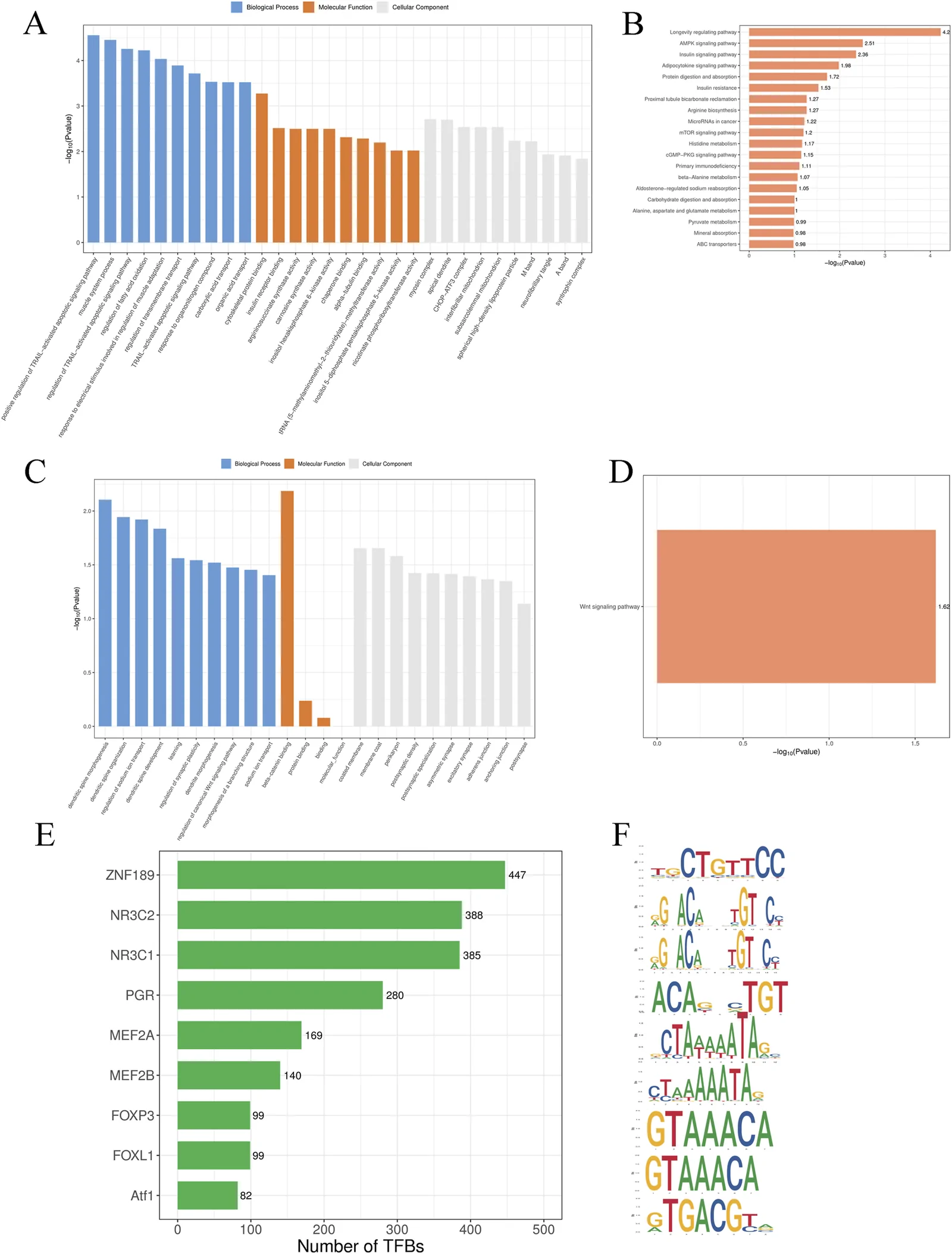
Differential gene function and transcriptional regulation. **(A)** GO enrichment of upregulated DEGs; **(B)** KEGG enrichment of upregulated DEGs; **(C)** GO enrichment of downregulated DEGs; **(D)** KEGG enrichment of downregulated DEGs; **(E)** Predicted transcription factors and their co-binding sites with star factors; **(F)** Motif types of transcription factors.

Integrated ATAC-seq and RNA-seq analyses identified 62 DEGs (59 upregulated, three downregulated) exhibiting correlated chromatin accessibility and expression changes, visualized through peak-gene association diagrams (Figures 5A,B).The top 20 DEGs ncluded 8 known muscle-development genes and 4 potential candidates (Figure 5C). Functional analysis revealed upregulated DEGs enriched in TRAIL-activated apoptotic signaling regulation, insulin receptor binding, and synaptic complexes (Figure 6A), with pathways including longevity regulation, AMPK signaling, insulin signaling, and cGMP-PKG-mediated homeostasis (Figure 6B). Downregulated DEGs showed enrichment in dendritic spine morphogenesis, β-catenin binding, and postsynaptic structure (Figure 6C), with Wnt/β-catenin signaling activation suggesting roles in synaptic plasticity and neurodevelopment (Figure 6D).

To further explore the potential upstream regulatory mechanisms of these DEGs, transcription factor enrichment analysis was performed. Several transcription factors, including *ZNF189*, *NR3C2*, *NR3C1*, *PGR*, and MEF family members, showed potential regulatory associations with the identified DEGs (Figure 6E). Motif enrichment analysis further identified conserved DNA-binding motifs among the accessible regulatory regions associated with these genes, suggesting that specific transcription factor binding events may contribute to the regulation of breed-specific muscle development (Figure 6F).

### 4.5 Validation of key myogenic genes confirms their roles in pectoral muscle development

**FIGURE 7:**
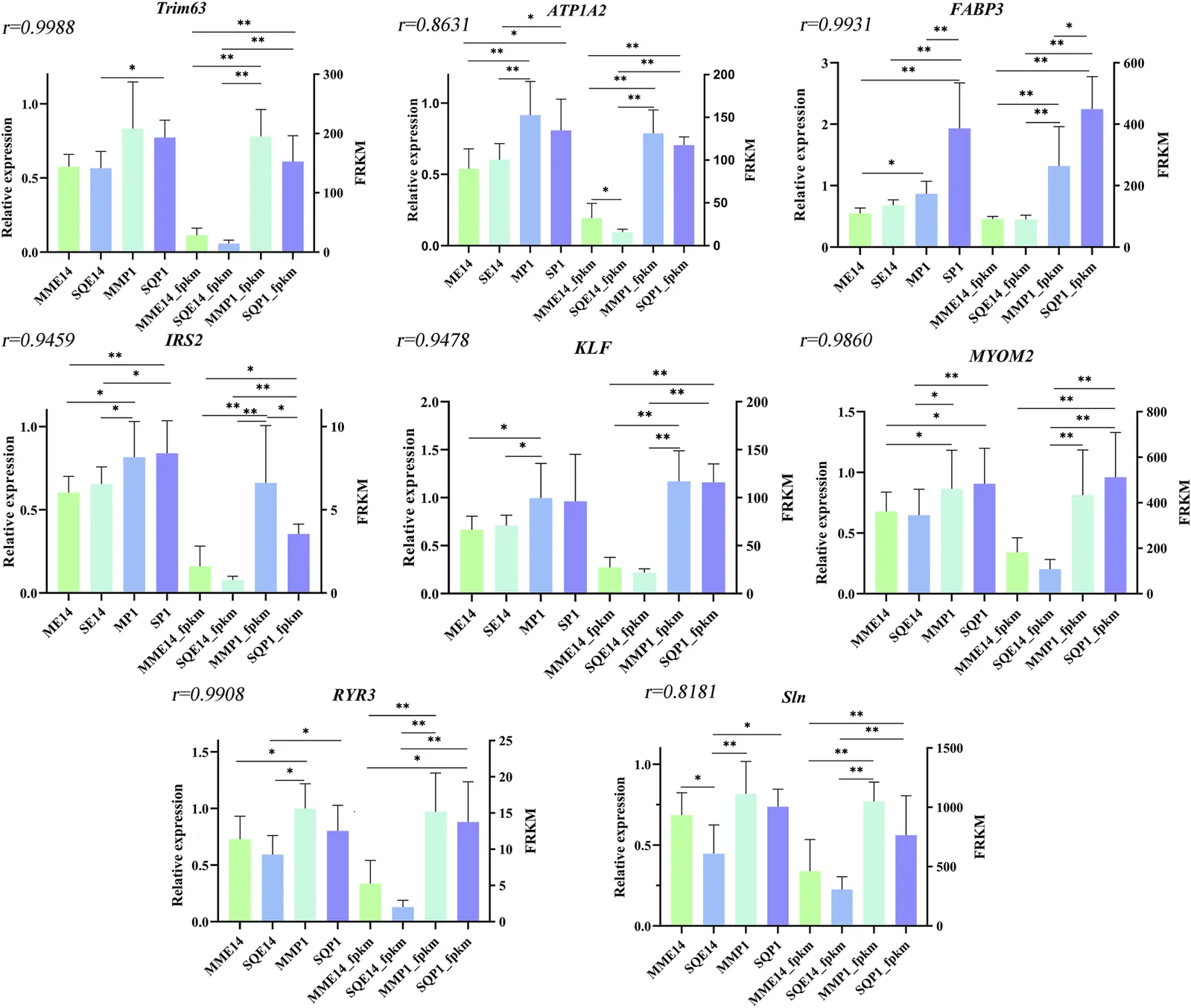
Validation of myogenic DEGs using RT-qPCR. RT-qPCR of *Sln, MYOM2, Trim63, Fabp3, ATP1A2, KLF15, IRS2* and *RYR3* in SQ and MM during E6-P1. *, *P* < 0.05; **, *P* < 0.01. The closer the absolute value of r is to 1, the stronger the linear correlation between the two variables.

Verification of pectoral muscle gene expression levels across the two pigeon breeds was conducted by analyzing a selection of eight myogenic genes, *Sln*, *MYOM2, Trim63, Fabp3, ATP1A2, KLF15, IRS2,RYR3*, that are essential for embryonic muscle development or displayed notable expression variations between breeds, were analyzed by RT-qPCR to precisely quantify their expression levels (Figure 7).

At P1, the relative expression of *Fabp3* in Shiqi pigeons was markedly elevated compared to that in European meat pigeons. Meanwhile, the expression of this gene at P1 was markedly elevated in both breeds compared to that at E14, but no notable disparity was detected between the two breeds at the E14 stage. The *ATP1A2* gene showed no significant difference between breeds during the same period, but its expression at P1 was markedly elevated compared to that at E14 in both breeds. The expression of the *Trim63* gene was also higher at P1 than at E14 in both breeds, although only Shiqi pigeons exhibited a significant difference between P1 and E14. The expression of the *IRS2* gene was markedly elevated at P1 than at E14 in both breeds. The expression of *KLF15* gene was higher at E14 than at P1, but only European meat pigeons showed a significant difference at E14 compared to the P1 stage in both breeds. The expression of the *MYOM2* gene at P1 was markedly elevated compared to that at E14. At P1, the expression of the *RYR3* gene was higher in European meat pigeons than in Shiqi pigeons, and it was also markedly elevated than that at E14 in both breeds. For Shiqi pigeon, the expression at P1 was markedly elevated compared to that at E14. The expression of the *Sln* gene at E14 was markedly elevated in European meat pigeons compared to Shiqi pigeons, and it was also notably higher than that at P1. It was also higher at P1 than in Shiqi pigeons, though without significant difference. The expression at P1 in both breeds was markedly elevated compared to that in Shiqi pigeons at E14. The results indicate that the expression trends of these genes, whether upregulated or downregulated, are consistent with the RNA-seq results (correlation coefficient (r) ≥ 0.8181).

### 4.6 Effects of *Sln* on proliferation and differentiation of pigeon embryonic myoblasts

mRNA expression profiling suggested that *Sln* may play a key role in pigeon muscle development (Supplementary Figure S3). To investigate its function, we modulated *Sln* expression in pigeon embryonic myoblasts during proliferation and differentiation stages using over-expression and interference techniques.

**FIGURE 8:**
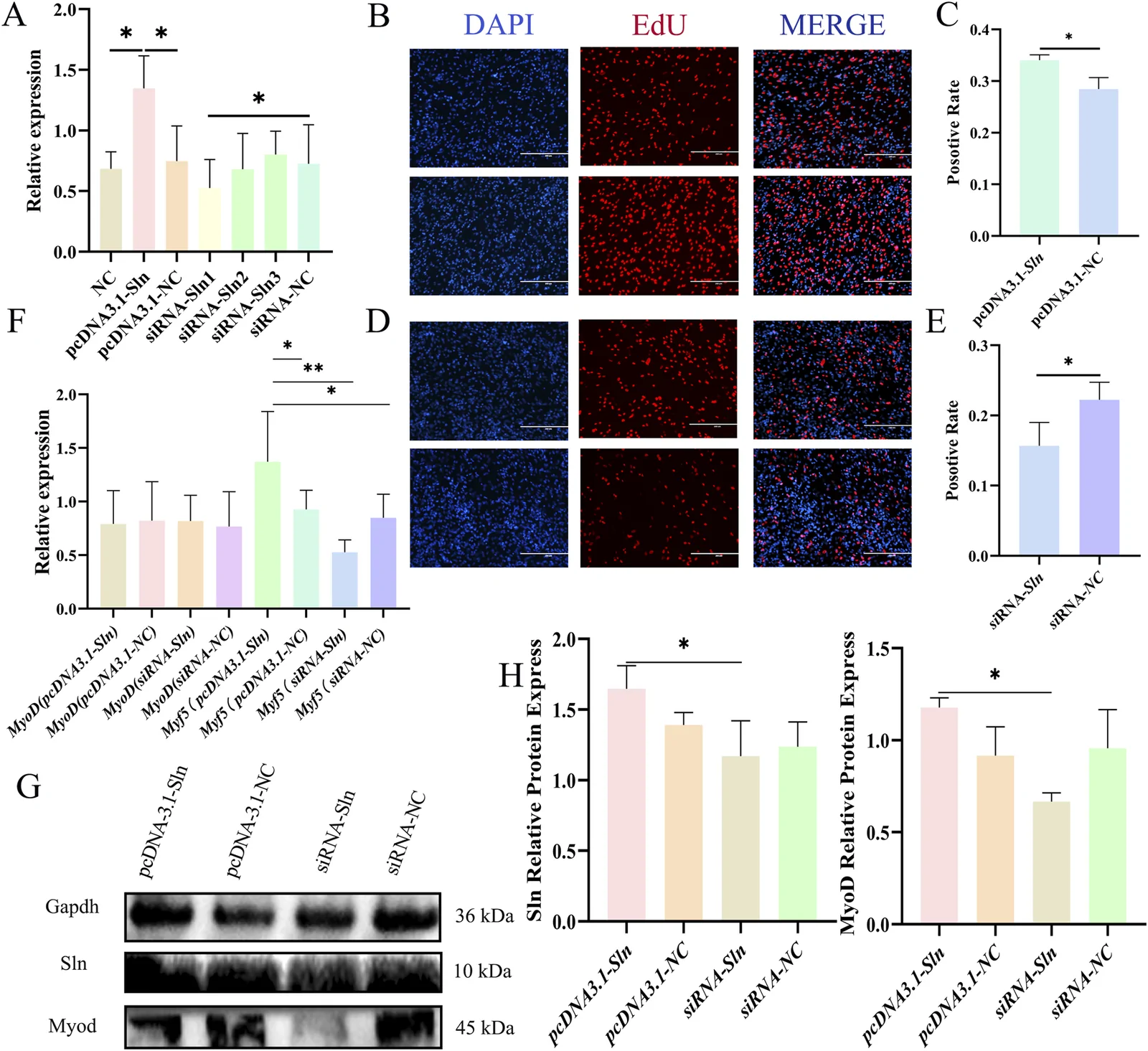
Effects of *Sln* over-expression and interference on proliferation of pigeon embryonic myoblasts. **(A)** Quantitative experimental validation of over-expression and interference efficiency; **(B)** EdU staining of pigeon embryonic myoblasts in the over-expression experiment, the upper panels represent the control group (pcDNA3.1-NC), and the lower panels represent the *Sln* over-expression group (pcDNA3.1-*Sln*); **(C)** Statistical analysis of the EdU-positive cell rate in the overexpression experiment; **(D)** EdU staining of pigeon embryonic myoblasts in the interference experiment; the upper panels represent the control group (siRNA-NC), and the lower panels represent the *Sln* interference group (siRNA-*Sln*); **(E)** Statistical analysis of EdU-positive cells in the interference group; **(F)** Quantitative analysis of marker gene expression during the proliferation stage; **(G)** Western blot analysis of proliferation-related proteins. Lanes 1–4 represent the pcDNA3.1-*Sln*, pcDNA3.1-NC, siRNA-*Sln*, and siRNA-NC groups, respectively. The pcDNA3.1-NC group served as the empty vector negative control for the Sln overexpression experiment, while the siRNA-NC group served as the non-targeting siRNA negative control for the Sln interference experiment. GAPDH was used as the loading control to verify equal protein loading among samples; **(H)** Densitometric quantification of target protein expression. The relative protein expression levels were calculated by normalizing the band intensity of Sln, MyoD to the corresponding GAPDH intensity. Band intensities were quantified using ImageJ software. Data are presented as mean ± SEM from three independent biological replicates. The primary antibodies used were anti-SLN (1:1000, Proteintech, 18395-1-AP), anti-MyoD (1:1000, Abcam, ab16148), anti-MyHC (1:10,000, Sigma, M4276), and anti-GAPDH (1:10,000, Abcam, ab181602). HRP-conjugated secondary antibodies were used according to the manufacturer’s instructions. Data are presented as mean ± SEM (n = 3). Statistical significance was determined using an unpaired two-tailed Student’s t-test (panels C, E and H) or one-way ANOVA followed by Tukey’s multiple comparison test (panels A and F). *P* < 0.05, *P* < 0.01.

During the proliferation stage, a set of *Sln* interference fragments was screened, and *Sln-1* was identified as the optimal fragment for subsequent functional experiments (Figure 8A). RT-qPCR analysis confirmed the successful regulation of *Sln* expression, showing that *Sln* mRNA expression was significantly upregulated in the pcDNA3.1-*Sln* group compared with the pcDNA3.1-NC group, whereas *Sln* expression was markedly decreased following siRNA-mediated knockdown compared with the siRNA-NC group (Figure 8A). EdU staining analysis further demonstrated that the EdU-positive cell rate was significantly increased in the *Sln* overexpression group (pcDNA3.1-*Sln*) compared with the empty vector control group (pcDNA3.1-NC), while *Sln* knockdown (siRNA-*Sln*) significantly reduced the proportion of EdU-positive cells compared with the negative control group (siRNA-NC) (Figures 8B–E). In addition, RT-qPCR analysis revealed that *Myf5* expression was significantly enhanced following *Sln* overexpression, whereas *Sln* interference suppressed *Myf5* expression (Figure 8F). Consistently, Western blot analysis showed that Sln protein levels were significantly increased in the overexpression group and decreased in the knockdown group, accompanied by similar changes in MyoD protein expression (Figures 8G,H). Collectively, these results demonstrate that *Sln* positively regulates myoblast proliferation during the proliferation stage and may promote the activation of myogenic regulatory programs (Figure 8).

**FIGURE 9:**
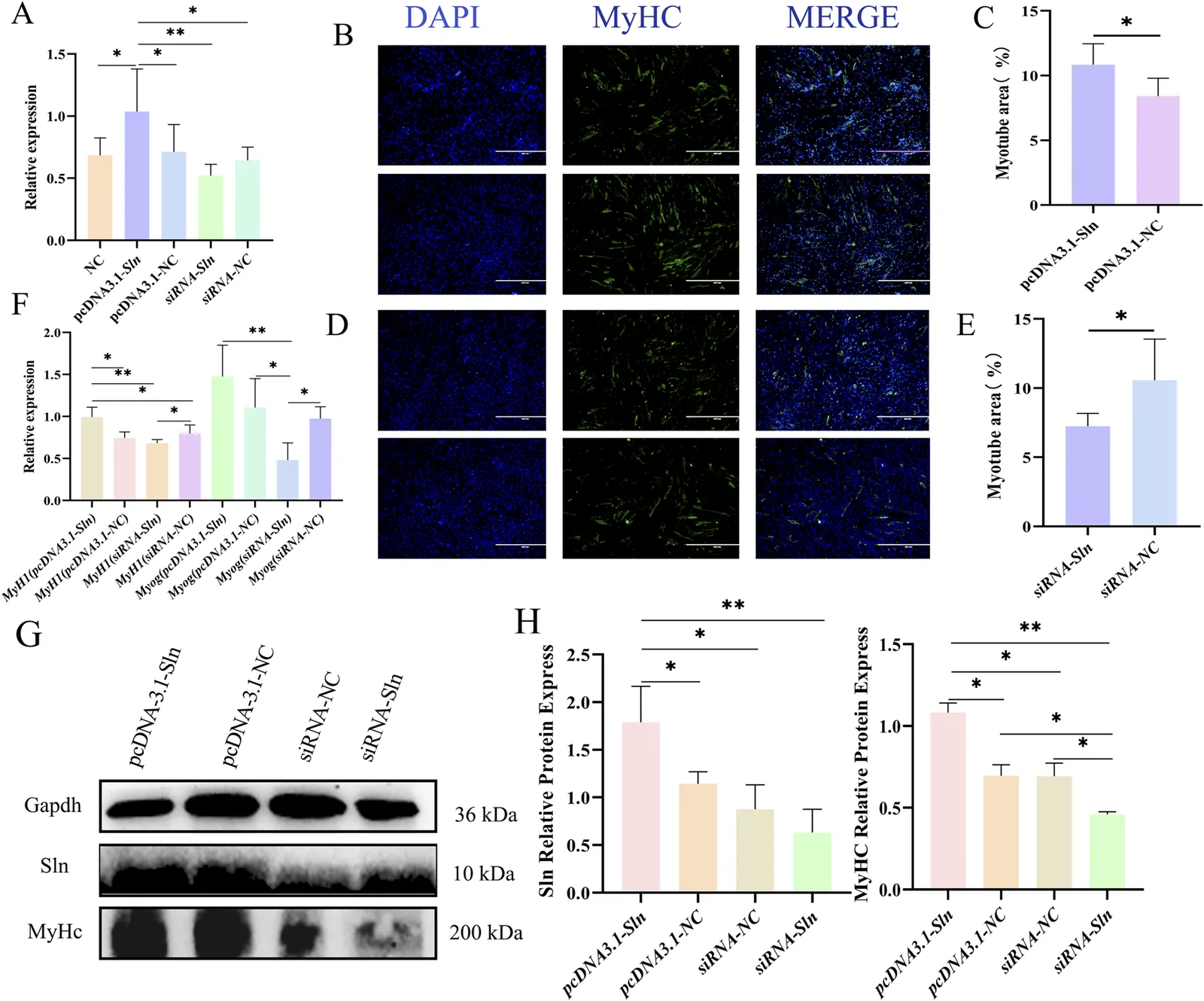
Effects of *Sln* over-expression and interference on differentiation of pigeon embryonic myoblasts. **(A)** Quantitative experimental validation of over-expression and interference efficiency; **(B)** MyHC immunofluorescence staining of pigeon embryonic myoblasts in the over-expression experiment; the upper panels represent the control group (pcDNA3.1-NC), and the lower panels represent the *Sln* over-expression group (pcDNA3.1-*Sln*); **(C)** Statistical analysis of MyHC fluorescence signals in the over-expression group; **(D)** MyHC immunofluorescence staining of pigeon embryonic myoblasts in the interference experiment; the upper panels represent the control group (siRNA-NC), and the lower panels represent the *Sln* interference group (siRNA-*Sln*); **(E)** Statistical analysis of MyHC fluorescence signals in the interference group; **(F)** Quantitative analysis of marker gene expression during the differentiation stage; **(G)** Western blot analysis of Sln and MyHC protein expression during myogenic differentiation. Lanes 1–4 represent the pcDNA3.1-*Sln*, pcDNA3.1-NC, siRNA-NC, and siRNA-*Sln* groups, respectively. GAPDH was used as the loading control to verify equal protein loading among samples; **(H)** Quantification of Sln and MyHC protein expression levels. The relative protein expression levels were calculated by normalizing the band intensity of Sln or MyHC to the corresponding GAPDH intensity. Band intensities were quantified using ImageJ software. The primary and secondary antibodies used for Western blot analysis were the same as those described for Figure 8. Data are presented as mean ± SEM (n = 3). Statistical significance was determined using an unpaired two-tailed Student’s t-test (panels C, E and H) or one-way ANOVA followed by Tukey’s multiple comparison test (panels A and F). *P* < 0.05, *P* < 0.01.

During the differentiation phase, the myotube fusion rate was significantly increased in the *Sln* overexpression group compared with the empty vector control group, whereas *Sln* knockdown resulted in a marked reduction in myotube formation (Figures 9B–E). RT-qPCR analysis confirmed the successful regulation of *Sln* expression, showing that *Sln* mRNA expression was significantly upregulated in the *Sln* overexpression group (pcDNA3.1-*Sln*) compared with the empty vector control group (pcDNA3.1-NC), while *Sln* expression was significantly decreased in the *Sln* interference group compared with the negative control group (siRNA-NC) (Figure 9A). Furthermore, RT-qPCR analysis revealed that *Sln* overexpression significantly increased the expression levels of *Myog* and *MyhC* during the differentiation stage, whereas *Sln* knockdown suppressed their expression (Figure 9F). Consistently, Western blot analysis showed that Sln and MyHC protein levels were significantly higher in the *Sln* overexpression group than in the control and knockdown groups, consistent with the quantitative analysis results (Figures 9G,H). Collectively, these findings demonstrate that *Sln* positively regulates myotube formation and promotes myogenic differentiation by enhancing the expression of key myogenic regulatory factors during the differentiation stage (Figure 9).

## 5 Discussion

Shiqi pigeons and European meat pigeons exhibit distinct pectoral muscle development patterns during embryogenesis, with pectoral muscle growth critically influencing meat quality, growth rate, and body size differences between the breeds. Our phenotypic analysis revealed that at P1, Shiqi pigeons had a markedly elevated proportion of 1–50 μm-diameter pectoral myofibers (*P* < 0.05), indicating breed-specific pectoral muscle development patterns during embryogenesis. Notably, our previous study on geese within our research group observed a similar pattern: the myofiber density of Wuzong geese during the embryonic stage was markedly elevated compared to that of Shitou geese (Zhang et al., 2023), suggesting a conserved association between body size and embryonic myofiber development rate in avian species, but there is a difference in the timing of divergence. Pigeons diverge at E14, while geese diverge at E23, which may be due to the larger embryo size and longer incubation period of geese. Further supporting this perspective, previous studies by other researchers across multiple breeds-including pigs (different body size breeds), chickens, and quails-have consistently shown that larger breeds/species exhibit slower embryonic muscle development (Cao et al., 2023; Shang et al., 2018; Zhang et al., 2019; Zhu et al., 2021), aligning with our current pigeon observations. Collectively, these cross-species findings reveal a conserved pattern: smaller-sized breeds tend to exhibit higher myofiber density or more rapid embryonic muscle development. This pattern provides a framework for interpreting the observed differences in pectoral muscle development between the smaller Shiqi pigeon and the larger European meat pigeon in our study. Specifically, we propose that the earlier and denser myofiber formation in Shiqi pigeons may establish a cellular basis for its distinct postnatal muscle growth trajectory and potentially influence meat quality traits.

Muscle development, a multifaceted physiological event, encompasses cellular regeneration, differentiation, and migration processes. The proper progression of this process depends on the precise regulation of related genes (Guo et al., 2022; Velleman, 2007). To identify potential genes and pathways associated with muscle growth and development, our research group previously performed transcriptome analysis on the pectoral muscles of Shiqi Pigeons and European Meat Pigeons (Li et al., 2023). Subsequently, this study conducted ATAC-seq and, based on the joint analysis of transcriptome data and ATAC-seq data, further explored the molecular mechanisms-such as gene pathways-that affect body size differences between the two pigeon breeds. By comparing chromatin accessibility-related genes and relevant differentially expressed genes, we found that the differences in gene expression between different developmental stages were significantly greater than those caused by breed variation. Breed differences mainly involved the coordinated regulation of proliferation capacity and metabolic regulation, and these regulations may be associated with immune or nutritional adaptation. Subsequently, 12 key candidate genes associated with breed differences were screened, including *Sln*. Further integrative analysis revealed that multiple candidate genes might participate in muscle development through long-range regulatory mechanisms. Further integrative analysis indicates that multiple candidate genes may participate in muscle development via long-range regulatory mechanisms. Precise regulation of muscle development-particularly long-range chromatin interactions (e.g., promoter-enhancer interactions)-primarily relies on dynamic changes in the three-dimensional genome structure. Notably, among the screened candidate genes, many contain key binding sites for essential proteins such as MEF2 and FOX family proteins. These factors may mediate the associations between enhancers and promoters (Freire-Pritchett et al., 2017; Szklarczyk et al., 2014). In summary, all eight genes ultimately selected for validation share a common feature: their involvement in long-range regulatory mechanisms, which reflects that these genes play a synergistic role in cell developmental processes or phenotype determination.

Many of these validated genes have been reported to be associated with the proliferation and differentiation of myoblasts. A similar study on pigeons has also identified *Fabp3* as a candidate gene involved in lipid metabolism. In addition, it has been shown to be associated with meat quality traits. Compared to White King pigeons, Shiqi pigeons, which have better meat quality, exhibit markedly elevated *Fabp3* expression levels, suggesting a regulatory role for this gene in meat quality (Ye et al., 2018; Zhang et al., 2013). In this study, the significant increase in *Fabp3* expression in Shiqi pigeons at the P1 stage (*P* < 0.05), combined with histological slice analysis results showing that Shiqi pigeons exhibited markedly elevated pectoral myofiber density and number than European meat pigeons during the early embryonic period, further supports the speculation that *Fabp3* might indirectly regulate myofiber number and density during embryonic development-potentially by modulating fat deposition.

Studies have shown that the *KLF15* gene is closely associated with muscle development. Research in chickens has shown that the *KLF15* gene inhibits abdominal fat deposition, a finding consistent with the markedly elevated expression of *KLF15* at the P1 stage compared to the E14 stage in the present study, which suggests its role in influencing myofiber development during early embryogenesis. Furthermore, polymorphisms in the *KLF15* gene have also been associated with carcass traits in domestic pigeons (Tian et al., 2022; Yin et al., 2016). Conversely, *IRS2* expression showed markedly elevated-expression in European meat pigeons at P1 (*P* < 0.05), consistent with its conserved role in avian muscle development and body weight regulation by mediating key metabolic signals in the insulin/IGF-1 pathway that stimulate growth,a mechanism that may explain the greater birth weight and faster growth rate observed in European meat pigeons compared to Shiqi pigeons (Dunislawska et al., 2021; Wang et al., 2020). Additionally, at E14 stage, *ATP1A2* expression was markedly upregulated in European meat pigeons (*P* < 0.05), mirroring its function in regulating glycolytic enzyme phosphorylation and nutrient partitioning to accelerate somatic development (Mármol Sánchez et al., 2020; Yang et al., 2025). In the process of screening genes affecting muscle development in Ningxiang pigs via ATAC-seq and RNA-seq, the *IRS2* gene was also identified (Tan et al., 2025). This finding may explain why European meat pigeon exhibit a faster growth rate than Shiqi pigeon during early embryonic stage, resulting in their higher myofiber density and number during this period.

The *TRIM63* gene encodes a ubiquitin ligase specifically expressed in muscle tissue, which participates in the modulation of skeletal muscle mass by mediating the ubiquitination and degradation of myofibrillar proteins. This study found that the expression of this gene was significantly upregulated in the late embryonic stage, suggesting its potential crucial role in skeletal muscle development (Hu et al., 2021; Peris-Moreno et al., 2020; Tian et al., 2022). *RYR3* encodes an endoplasmic reticulum calcium release channel that participates in the fine-tuning of learning and memory formation as well as muscle contraction by regulating intracellular calcium homeostasis. In this experiment, the expression of *RYR3* also showed a significant upregulation trend in the late embryonic stages, implying its potential involvement in metabolic regulation and muscle development during the embryonic period through the calcium signaling pathway (Hu et al., 2021; Sachs et al., 2019; Tian et al., 2022). The *TRIM63* and *RYR3*, both significantly upregulated in the P1 stage, may jointly contribute to the observed differences in pectoral myofiber density and number between Shiqi pigeon and European meat pigeon-with *TRIM63* potentially modulating muscle mass through protein turnover and *RYR3* possibly influencing development via calcium signaling pathways.

Among genes exhibiting significant stage-specific expression differences, *Sln* showed notable expression variations across developmental stages in both pigeon breeds. Specifically, at the E14 stage, the *Sln* gene expression was markedly elevated in European meat pigeons compared to Shiqi pigeons, while Shiqi pigeons exhibited a markedly elevated myofiber density. Furthermore, tissue-specific validation confirmed that *Sln* is highly expressed only in muscle tissue. It can be speculated that this gene, which critically regulates muscle thermogenesis and modulates metabolic functions, may be a factor influencing the differences between the two breeds during embryonic developmental stages, particularly in the observed characteristics that European meat pigeons exhibit markedly decreased myofiber number and diameter compared to Shiqi pigeons (Conte et al., 2023; Manring et al., 2014; Periasamy et al., 2017). Furthermore, a multi-omics study conducted on the longissimus dorsi muscle of Luchuan pig identified *Sln* as a key gene, which was significantly upregulated during the late stages of embryonic muscle development, indicating its role as a regulatory factor (Yuan R, 2021). Therefore, this gene has been prioritized as a key gene of focus, with verification performed on myoblasts from the pectoral muscle of pigeon embryos.

Cellular functional assays (EdU and MyHC staining) confirmed that *Sln* positively regulates pigeon myoblast proliferation and differentiation. The successful over-expression and knockdown of *Sln* were validated (Figures 9A–E). Over-expression of *Sln* upregulated *Myf5* expression during the proliferation stage, while *MyoD* levels remained unchanged, likely due to functional compensation between *MyoD* and *Myf5* (Wang et al., 1996). During differentiation, *Sln* over-expression significantly elevated *Myog* and *MyHC* expression. Western blot further validated Sln protein expression and revealed its synergistic regulation of MyoD (proliferation phase) and MyHC (differentiation phase). During cell differentiation or treatment, the peak level of *MyoD* mRNA typically occurs at an early time point, whereas its protein peak appears later (e.g., at 48 h) due to a longer half-life. If sampling is performed at an intermediate time point when the protein level is still high but the mRNA level has already declined, a phenomenon of “no difference in mRNA but a difference in Western blot results” will be observed. Furthermore, an increase in *MyoD* protein without a corresponding increase in mRNA may indicate that some cells have entered a more mature differentiation stage, maintaining the myogenic program through post-translational stabilization mechanisms. Among these mechanisms, activation of the AKT/mTOR signaling pathway can enhance *MyoD* translational efficiency or suppress its ubiquitin-mediated degradation, leading to protein accumulation without changes in mRNA levels (Chen et al., 2025; Cheon et al., 2024; Zhang et al., 2015).

Quantitative analyses indicated the involvement of *Myog* and *MyoD*, potentially through synergistic interactions among myogenic regulatory factors-a mechanism previously reported during embryonic skeletal muscle development in mice and humans (Geetha-Loganathan et al., 2005; Otto et al., 2006; Mavalli et al., 2010). Conversely, after *Sln* interference, indicators such as the myotube fusion index were significantly reduced, highlighting the critical role of this gene in the terminal differentiation of myoblasts. This functional impairment may be attributed to the suppressed expression of MRFs (Hu et al., 2026; Li et al., 2025; Ma et al., 2025). *Sln* may act as a novel upstream regulator that cooperatively activates the expression of *MyoD* and *Myog* at the transcriptional or epigenetic level through interactions with transcriptional co-factors or signaling pathways.

Compared to single-omics studies-such as those in transcriptome research of different duck breeds (Cao et al., 2023; Du et al., 2022; Gong et al., 2021), which typically identifies DEGs and pathways-integrated ATAC-seq and RNA-seq analysis reveals changes in chromatin accessibility across chromosomes. By integrating ATAC-seq open regions with RNA-seq DEGs, researchers can more systematically investigate the transcriptional regulation that is linked to chromatin accessibility, thus providing a broader understanding of gene expression control to better clarify the process and regulation of myogenesis (Buenrostro et al., 2015; Li et al., 2024; Liu et al., 2019). This comprehensive research approach has been used to compare muscle development differences among breeds with varying body sizes. However, a study on broilers showed results markedly different from those of the present study: fast-growing broilers typically significantly upregulate muscle repair-related genes (likely because rapid growth tends to cause muscle microdamage), whereas our analysis of pigeons revealed that these muscle repair-related genes were not significantly upregulated. This discrepancy may be related to the slower muscle development rate of squabs compared to precocious broilers (Gu et al., 2024; Ren et al., 2024; Zhu et al., 2023).

In similar studies conducted by our research group, two breeds with significant body size differences-Shitou geese and Wuzong geese-were examined. During the late embryonic stage, the myofiber density of Shitou geese was significantly greater than that of Wuzong geese. It was found that Shitou geese possess significantly more unique genes, which are markedly enriched in processes related to muscle development (Shao et al., 2025; Zhang et al., 2021). This result aligns with the trend observed in this study large-sized pigeon breeds tend to activate more DEGs at critical pectoral muscle development stages. Specifically, at E14, this study identified more DEGs from the pectoral muscle samples of European Meat Pigeons, with most showing markedly elevated-expression levels than their counterparts in Shiqi Pigeons. Further analysis revealed stronger gene enrichment in muscle development-related pathways in European Meat Pigeons, while Shiqi Pigeons exhibited relative advantages in certain energy metabolism regulatory pathways. The differences in gene expression and pathway enrichment between these two pigeon breeds may account for variations in myofiber density, quantity, and embryonic pectoral muscle development rates. However, it is noteworthy that in geese at E23, large body-sized geese exhibit significantly higher myofiber density and quantity than small body sized geese; yet, the small body sized pigeons in this study (at E14) showed higher myofiber quantity and density than geese at the same stage (E23 in geese). This discrepancy may stem from divergence in their developmental strategies: as precocial birds, geese prioritize rapid muscle development during embryogenesis to support autonomous activity after hatching, thus allocating resources preferentially to muscle growth. In contrast, as altricial birds, pigeons prioritize visceral and nervous system development during embryogenesis, resulting in a relatively delayed muscle development rhythm but a higher unit density advantage at early stages.

A consistent trend observed in this study is that large-bodied pigeon breeds often have more DEGs activated at key pectoral muscle development stages, such as E14, which aligns with the results reported here (Figure 3F). Specifically, at stage E14, this study screened out more DEGs from European meat pigeon pectoral muscle samples, with most of these DEGs exhibiting markedly elevated-expression levels compared to the corresponding genes in Shiqi pigeons. Further analysis showed that European meat pigeons had greater gene enrichment in muscle development-related pathways, while Shiqui pigeons had comparative advantages in some energy metabolism-regulating pathways. The differences in gene expression and gene enrichment pathways between these two pigeon breeds may be the reason for the variations in their myofiber density and number, as well as the speed of pectoral muscle development during embryonic stages. This study, through integrating multi-omics analysis, revealed that the expression differences of key genes such as *Sln* and *Fabp3* in the pectoral muscle during embryonic development of Shiqi pigeons and European meat pigeons are the core molecular basis for the body size differences between the two pigeon breeds. Specifically, during critical stages of embryonic development, there are significant differences in the expression profiles of genes such as *Sln* and *Fabp3* between European meat pigeons (large-bodied) and Shiqi pigeons (medium-bodied). By regulating myoblast proliferation and differentiation as well as the number of myofibers in the early embryonic stage, this ultimately leads to divergent pectoral muscle developmental trajectories during embryogenesis-with the pectoral muscle of European meat pigeons entering a rapid growth phase earlier to lay the foundation for their large body size, while those of Shiqi pigeons develop at a relatively slower pace.

Nevertheless, these findings are constrained by methodological limitations, most notably the analysis being confined to select embryonic stages such as P1, which fails to encompass the full continuum of the dynamic developmental trajectory from embryogenesis to sexual maturity, and this limits the understanding of the dynamic evolution of regulatory networks. Secondly, although potential transcription factors were inferred by integrating chromatin accessibility and transcriptome data, the direct regulatory mechanisms between these factors and downstream genes has not been experimentally validated. From an application perspective, the key genes identified in this study provide a foundation for developing molecular breeding markers, which could be utilized for marker-assisted selection in pigeons and other poultry species, thereby accelerating the breeding process of high-quality meat pigeons. Specifically, the core application direction is to leverage these screened genes to enhance traits in Shiqi pigeons-known for their good meat quality-by employing techniques including gene editing (for example, CRISPR-Cas9), marker-based molecular selection (MAS), and genome-wide selection (GS) to promote larger body size and faster early growth rates, thereby optimizing their overall production value.

## 6 Conclusion

**FIGURE 10:**
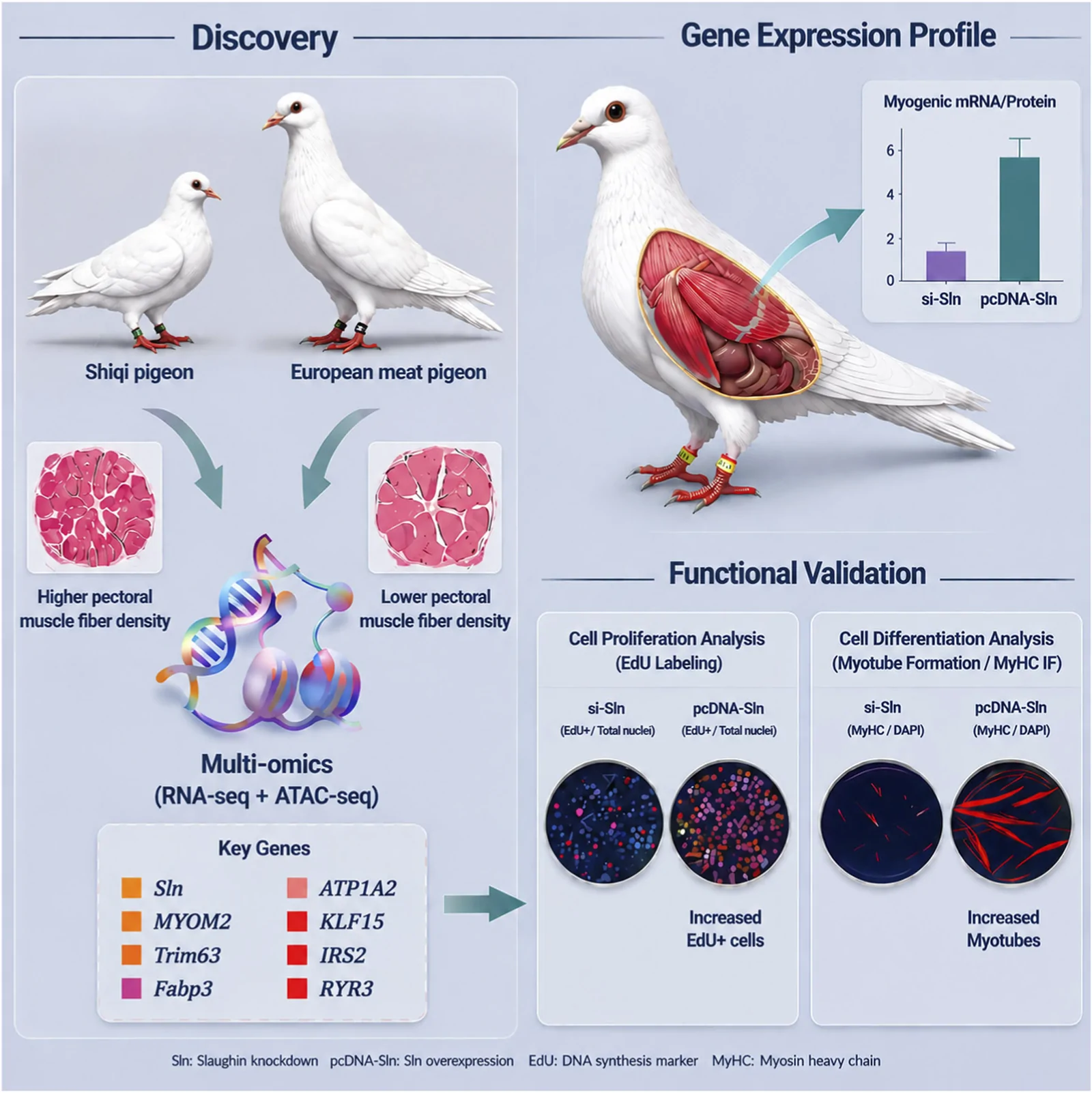
Schematic diagram illustrating the molecular mechanisms regulating breast muscle development in meat pigeons. Based on integrated multi-omics analysis and functional validation, this study identifies key genes, such as *Sln*, through phenotypic comparison and multi-omics (RNA-seq and ATAC-seq) data screening, and validates their role in promoting myoblast proliferation and differentiation at both gene expression and functional levels, revealing critical molecular pathways affecting pectoral muscle myofiber density in large and small body size pigeon breeds during the embryonic stage.

In summary, Shiqi pigeon pectoral muscle at P1 stage has significantly greater myofiber density than European meat pigeons. Integrated RNA-seq and ATAC-seq identified 62 candidate genes and several pathways regulating early myogenic gene expression. RT-qPCR validation of eight selected genes (*Sln, MYOM2, Trim63, Fabp3, ATP1A2, KLF15, IRS2, RYR3*) matched transcriptome results. Tissue expression profiling across liver, intestine, heart, pectoral, and leg muscles selected *Sln* for functional validation (Figure 10). Cellular and quantitative data indicate that *Sln* positively regulates the proliferation and differentiation of pigeon myoblasts through *Myf5* (proliferation) and *Myog*/*MyH1* (differentiation). Western blot analysis further demonstrated that *Sln* modulates the protein expression of myogenic factors such as MyoD and MyHC. This study reveals breed differences in embryonic chromatin accessibility and gene expression, providing a theoretical basis for elucidating molecular mechanisms of skeletal muscle development in different-sized pigeons and supporting future molecular marker-assisted breeding.

## Data Availability

The ATAC-sequencing data generated and analyzed in this study have been deposited in the NCBI Sequence Read Archive (SRA) under the accession number (PRJNA1244945). The raw RNA-seq data have been submitted to the SRA database under the accession number (PRJNA1005959).
